# Development and validation of the Japanese version of the obsessive-compulsive inventory

**DOI:** 10.1186/1756-0500-7-306

**Published:** 2014-05-20

**Authors:** Ryotaro Ishikawa, Osamu Kobori, Eiji Shimizu

**Affiliations:** 1 Research Fellow of the Japan Society for the Promotion of Science, Japan; 2 Centre for Forensic Mental Health, Chiba University, Japan; 3 Department of Cognitive Behavioral Physiology, Chiba University Graduate School of Medicine, Japan; 4 Research Centre for Child Mental Development, Graduate School of Medicine, Chiba University, Japan

## Abstract

**Background:**

The Obsessive-Compulsive Inventory (OCI) was designed to evaluate the severity of obsessive-compulsive symptoms in both clinical and non-clinical samples. The aim of the study was to develop a Japanese version of this scale (OCI-J) and validate it in both non-clinical and clinical Japanese samples.

**Findings:**

In Study 1, the OCI-J, the Maudsley Obsessional Compulsive Inventory (MOCI), and measures of anxiety and depression were administered to 150 undergraduate students (non-clinical sample) in order to investigate the internal consistency and convergent validity of the OCI-J. Furthermore, 118 non-clinical participants completed the OCI-J after a 2-week interval to determine the test-retest reliability. In Study 2, OCD participants (n = 35), anxiety control participants with panic disorder (n = 22), and healthy control participants (n = 37) completed the OCI-J in order to test its clinical discrimination ability.

Correlational analysis indicated moderate to high correlations between the subscales and total scores of the OCI-J and MOCI. In addition, the OCI-J and its subscales demonstrated satisfactory test-retest reliabilities. Finally, the OCI-J showed good clinical discrimination for patients with OCD from healthy and anxiety controls.

**Conclusions:**

The OCI-J is a valid and reliable instrument for measuring OCD symptoms in both clinical and non-clinical samples of Japanese.

## Findings

The aim of this study was to develop a Japanese version of this scale (OCI-J) and validate it in both non-clinical and clinical Japanese samples. Our findings demonstrated that the OCI-J is a valid and reliable instrument for measuring OCD symptoms in both clinical and non-clinical samples of Japanese. The availability of the OCI-J provides researchers with an additional measure for assessing the severity of OCD symptoms.

### Background

Obsessive-compulsive disorder (OCD) is a chronic psychiatric illness with a mean lifetime prevalence of 2–3% in the general population [1]. An anxiety-based disorder, OCD is characterized by persistent, intrusive, and distressing obsessions (persistent thoughts, impulses, or images) or compulsions (repetitive, excessive behaviors or mental acts) [2].

In Japan, there is a national prevalence of about 2% for OCD, as in the US [3]. In one study, researchers administered the Yale-Brown Obsessive Compulsive Scale (Y-BOCS) symptom checklist [4] to 343 Japanese OCD patients to examine whether symptom dimensions were stable across cultures [5]. They found that the OCD symptom structure has substantial transcultural stability across Western and Eastern cultures, suggesting that OCD is mediated by universal psychobiological mechanisms [5]. Although several questionnaires have been developed that evaluate the severity of OCD symptoms in the Japanese population, such as the Japanese versions of the Y-BOCS [6] and Maudsley Obsessive-Compulsive Inventory (MOCI-J) [7], none are suitable for quick, effective clinical assessments. In particular, the Y-BOCS, one of the most commonly used scales in OCD research, uses a semi-structured interview format, consisting of 10 core items that assess time spent on obsessions or compulsions, resistance, interference, distress, and control [4,6]. The scale yields three severity scores: obsessions, compulsions, and an overall score. Furthermore, it possesses a 67-item symptom checklist for an accurate assessment of symptoms. This scale has excellent psychometric properties and is useful in research on treatment outcomes [8], but it has three notable limitations that prevent it from being effective for clinical settings. First, several studies have indicated that this instrument may not correlate well with other measures of obsessive-compulsive symptoms. For example, Goodman et al. [4] found that Y-BOCS scores did not consistently correlate with the MOCI [9]. In addition, since the Y-BOCS has moderate correlations with depression and anxiety measures [4,10], its discriminant validity is low [11]. Second, due to its interview-based format, it can be time-consuming and expensive to administer. As an interview, it requires trained interviewers, and interviewer reliability must be established to ensure accurate results. The 10 core items do not contain information on the specific content of obsessions and compulsions. Although this information can be obtained from the YBOCS checklist, the checklist has a large number of items, which makes it much more difficult to quickly identify the nature and severity of patients’ symptoms [12]. The Y-BOCS symptom checklist includes a dichotomous list of subtypes, but it does not provide a continuous measure of OCD symptom dimensions. Therefore, Y-BOCS scores are less affected by the subtypes of OCD; for example, patients with “mixed-OCD” may have low Y-BOCS scores, even though they suffer from several OCD symptoms. Thus, the Y-BOCS is difficult to use in clinical settings [11]. A self-report version of the Y-BOCS was created by Baer [13]; this version assesses the same 15 categories as the interview version. Respondents are asked to report to what extent they have experience with items such as *I have violent or horrific images in my mind* or *I am concerned with dirt or germs.* However, Wu et al. [14] raised a number of critical points regarding the symptom checklist of the self-report Y-BOCS, including the validity of the rationally based assignment of symptoms to categories, the inadequacy of the self-report format to distinguish OCD from non-OCD samples, and issues surrounding the wording of items [14,15].

Unlike the interview-based Y-BOCS, the MOCI [9] is a self-report measure, and a Japanese version of the MOCI (MOCI-J) has been developed [7]. It consists of 30 true/false items and has satisfactory test–retest reliability and internal consistency [7,16]. Factor analysis has revealed four subscales: Cleaning, Checking, Slowness, and Doubting [9]. However, the MOCI has two limitations [11,12]. First, the dichotomous true–false format makes the scale less sensitive overall, as it can only assess the severity of specific symptoms, and it may only be effective in assessing changes in severity post-treatment. Second, some of its items are not directly linked to OC symptoms. The MOCI-J does not provide an adequate assessment of obsessional rumination [17]. In addition, this scale primarily assesses compulsive rituals and overemphasizes Cleaning and Checking rituals to the exclusion of other types of neutralizing activities [11]. Thus, although the inclusion of the four subscales appears to better address the heterogeneity of OCD, the subscales capture only a subset of OCD symptoms [11][12]. Another questionnaire, the Padua Inventory [18] contains 60 items, each rated on a 0–5 scale, that describe common obsessions and behavioral compulsions. The Padua Inventory yields four factors: Impaired Mental Control, Contamination, Checking, and Loss of Control of Actions [18]. The questionnaire has adequate internal consistency, test-retest reliability, and discriminate validity [11,18]. However, the instrument has difficulty differentiating obsession and worry [19]. Furthermore, the Padua Inventory does not include some categories of obsessions and compulsions, such as neutralizing and hoarding [12,20].

The Obsessive-Compulsive Inventory (OCI) is another self-report scale for measuring OC symptoms [12]. This scale has 42 items (e.g., *I avoid using public toilets because I am afraid of disease or contamination*), each of which is rated on a five-point Likert scale corresponding to frequency of symptoms in the past month and severity of distress (e.g., 0 = “not at all distressed” to 4 = “extremely distressed”). The full scale yields a total possible score of 168. We believe that the OCI is a more advantageous measurement than the other scales discussed for three reasons.

First, the OCI is a more comprehensive instrument than the Y-BOCS, MOCI, or Padua Inventory because it contains seven subscales, which allows it to capture the considerable heterogeneity of obsessions and compulsions [12]. These subscales include Washing (eight items), Checking (nine items), Mental Neutralizing (six items), Obsessing (eight items), Ordering (five items), Hoarding (three items), and Doubting (three items).

In addition, unlike the Y-BOCS, administration of the OCI does not require trained interviewers. Therefore, the OCI covers a wide range of OC phenomena in a format that is easy to administer and can be used to assess not only obsessions and compulsions in groups with diagnosable OCD, but also OC thoughts and behaviors in the general population [12,21]. Foa et al. [12] reported good to excellent internal consistency for both the full scale and the subscales for patients with OCD, and found that the scale had good to excellent test-retest reliability for OCD patients across two weeks. The OCI also demonstrates excellent discriminant validity because OCD patients reported greater distress on the OCI than other-anxious controls (i.e., people with posttraumatic stress disorder or generalized social phobia). Finally, because the OCI total scores were positively associated with the total scores of the MOCI, the OCI was shown to have satisfactory convergent validity [12]. The psychometric properties of the OCI and its subscales have also been examined in a non-clinical student sample [21], which indicated a high internal consistency and good test-retest reliability for the total scale and each subscale. Simonds et al. [21] also found that the OCI had good convergent validity with the MOCI [9]. However, there has been no empirical study using the OCI in Eastern cultures.

The Obsessive–Compulsive Inventory—Revised (OCI-R [22]) is a brief (18-item) adaptation of the 42-item OCI [12]. In addition to yielding a total score, the OCI-R has six subscales: Washing, Checking, Ordering, Obsessing, Hoarding, and Neutralizing. The OCI-R has excellent psychometric properties in a mixed sample of patients with obsessive–compulsive disorder and other anxiety disorders.

Peng et al. [23] translated the OCI-R [22] into Chinese, and administered it to a non-clinical sample and patients with OCD. The study suggested that the Chinese version demonstrated good validity. Chasson et al. [24] evaluated the psychometric properties of the translation [23], and found strong evidence for its test-retest reliability and construct validity [24]. However, in a non-clinical sample, the internal consistency (Cronbach’s alpha) for Neutralizing (alpha = .34) and Checking (alpha = .65) subscales of OCI-R were inadequate [22]. Hajcak et al. [25] also found that the hoarding (alpha = .65) and neutralizing (alpha = .47) subscales of OCI-R did not have adequate internal consistency. In the study by Peng et al. [23], which included 209 Chinese undergraduates and 56 individuals with OCD, the majority of the coefficient alphas for the subscales of OCI-R were not strong (Washing = .64, Obsessing = .77, Hoarding = .66, Ordering = .63, Checking = .61, and Neutralizing = .53). Thus, the internal consistency of the subscales of OCI-R in non-clinical sample are not stable, as compared to OCI subscales which indicated a high internal consistencies (alpha = .78 to .95) in non-clinical sample [21].

In summary, the OCI is quick, easy to administer, and can assess a range of obsessional and compulsive behaviors. In addition, the scale has a strong evidence for its internal consistency, test-retest reliability, and construct validity in both clinical and non-clinical samples. This makes it a useful supplement to existing measures that are capable of assessing both clinical and non-clinical OC phenomena. The OCI could be especially useful in providing a comprehensive assessment of the severity of a range of OC phenomena in non-clinical samples, which could then serve as analogues for those with clinical OCD. For these reasons, a comprehensive yet brief self-report measure of OCD symptoms, such as the OCI, would be useful for assessing OCD in Japan.

The purpose of this study was to validate a Japanese version of the OCI (OCI-J) in non-clinical and clinical samples. To achieve this, we examined its convergent validity and test-retest reliability. We also examined the scale’s ability to discriminate between individuals with OCD and individuals with another anxiety disorders by comparing the scores of three key groups: OCD patients, healthy controls (non-clinical participants), and anxiety controls with panic disorder. We predicted that OCD participants would have much higher scores on the OCI than would the participants in the other two groups.

## Study 1

### Purpose

The aims of Study 1, which used a non-clinical sample, were to investigate the internal consistency of the OCI-J; confirm the convergent validity of the OCI-J by determining the correlation with another measure of OC symptoms (MOCI-J) and other measures of psychopathology, such as symptoms of depression and anxiety; and determine the test-retest reliability of the OCI-J.

### Methods

The data of 150 Japanese undergraduates (68.3% female; age range: 18–50 years, *M* = 20.60, *SD* = 3.94) were used to estimate the convergent validity of the OCI-J. In addition, 118 non-clinical Japanese undergraduates (56.3% male; age range: 18–27 years; *M* = 19.62, *SD* = 1.12) participated in establishing the test-retest reliability; these participants were different from those who took part in testing the convergent validity.

Information about this study was provided through handouts and oral presentations given in lecture rooms at the university sites. Interested students were asked to contact the researchers, and those who did received and completed the questionnaire. This study was approved by the ethics committee of, the Graduate School of Medicine, Chiba University (receipt number, 1122).

### Measurements

#### Translation and adaptation of the OCI–J

We developed the Japanese version of the OCI through a back-translation procedure. The second author translated the original OCI into Japanese. An independent bilingual translator (a Japanese university student with a psychology major who had been living in the U.K. for more than ten years) then completed a back translation. Subsequently, we, along with an author of the original OCI, compared these versions and amended the Japanese version accordingly. The initial OCI-J consisted of 42 items. In the original OCI, participants rated each item in terms of the frequency of obsessions and compulsions as well as the distress related to these phenomena on a five-point Likert scale. However, Foa et al. and others have repeatedly found a high correlation between the distress and frequency total scores, suggesting that these two scales are redundant [11,22,26,27]. Therefore, we elected to include only the distress measure in the OCI-J.

#### MOCI-J

The MOCI [9] is a 30-item self-report scale (e.g., I *worry unduly about contamination if I touch an animal*) that uses a true-false response format. This scale yields a total obsession score and four subscale scores: Cleaning, Checking, Slowness, and Doubting [9]. The Japanese version of the MOCI (MOCI-J) was developed and confirmed to have adequate reliability and validity by Yoshida et al. [28].

#### State-Trait Anxiety Inventory (STAI)

Anxiety symptoms were assessed using Spielberger’s STAI [29], a 40-item self-report scale, with 20 items assessing state anxiety (e.g., *I am presently worrying over possible misfortunes*) and 20 assessing trait anxiety (e.g*., I am a steady person*). All items are rated on a four-point scale: 0 (almost never) to 3 (almost always). The Japanese version of the STAI was developed and validated by Nakazato and Mizuguchi [30]. Following previous studies comparing the OCI and the STAI [12], we used only the trait scale of the STAI to investigate how the OCI-J correlates with trait anxiety.

#### Center for Epidemiologic Studies Depression (CES-D) scale

The CES-D is one of the most widely used self-report instruments for measuring current depressive symptomatology and identifying possible depressive disorders (e.g., *I am bothered by things that usually don’t bother me*) [31]. All items are rated on a four-point scale: 1 (rarely or none of the time), 2 (some or a little of the time), 3 (occasionally or a moderate amount of time), and 4 (most or all of the time). This scale was created by selecting items from various depression scales (e.g., the Beck Depression Inventory [32]) assumed to measure the most important components of depressive symptomatology, including depressed mood, feelings of guilt and worthlessness, feelings of helplessness, loss of appetite, sleep disturbances, and psychomotor retardation [31]. The Japanese version of the CES-D was developed and validated by Shima et al. [33]. We used only the total score in our analysis.

## Results

### Mean, standard deviation, and internal consistency

The mean, SD, and internal consistency (Cronbach’s alpha) values of the scales are presented in Table 1. The Cronbach’s alpha values ranged from .80 to .96, indicating good internal consistency.

**Table 1:** Means, SDs, and internal consistency of the scales compared in this study (N = 150 Japanese undergraduates)

	**Mean (SD)**	**Alpha**
OCI-J Total	30.21 (23.51)	.96
Washing	5.68 (3.39)	.90
Checking	6.70 (6.21)	.91
Doubting	2.78 (2.33)	.89
Ordering	3.04 (3.11)	.82
Obsessing	6.71 (6.21)	.91
Hoarding	1.63 (2.64)	.80
Neutralizing	3.32 (3.11)	.85
MOCI-J Total	8.89 (4.52)	.71
Cleaning	2.86 (1.90)	.64
Checking	2.71 (1.86)	.65
Doubting	2.95 (1.64)	.58
Slowness	2.18 (1.51)	.54
STAI	30.51 (10.39)	.71
CES-D	10.36 (7.21)	.79

CES-D = Center for Epidemiological Studies Depression scale; MOCI-J = Maudsley Obsessive-Compulsive Inventory, Japanese version; OCI-J = Obsessive-Compulsive Inventory, Japanese version; STAI = State-Trait Anxiety Inventory.

### Correlations between variables

The correlation coefficients between the OCI-J total scores and the subscale scores are presented in Table 2. Consistent with our predictions, the total and most of the subscale scores (Washing, Checking, Doubting, Ordering, Obsessing, and Neutralizing) of the OCI-J were significantly correlated. Hoarding was significantly correlated with the OCI-J total, Checking, Doubting, Ordering, Obsessing, and Neutralizing, but was not correlated with Washing.

**Table 2:** Correlations between the OCI-J total scores and the OCI-J subscales (N = 150 Japanese undergraduates)

	**Washing**	**Checking**	**Doubting**	**Ordering**	**Obsessing**	**Hoarding**
OCI	.62***	.57***	.42***	.60***	.68***	.31**
Washing	-	.81***	.46***	.56***	.67***	.12
Checking	.81***	-	.48***	.53***	.67***	.33***
Doubting	.46***	.48***	-	.49***	.55***	.22**
Ordering	56***	53***	.49***	-	.65***	.31**
Obsessing	.67***	.67***	.55***	.65***	-	.26**
Hoarding	.12	.33***	.22**	.31**	.26**	-
Neutralizing	.65***	.59***	.40***	.51***	.66***	.23**

**p < .01, ***p < .001.

The correlation coefficients between the OCI-J, MOCI-J, STAI, and CES-D are presented in Table 3. Consistent with our predictions, the total and subscale scores of the OCI-J were significantly correlated with the MOCI-J, STAI, and CES-D.

**Table 3:** Correlations between the OCI-J and MOCI-J (N = 150 Japanese undergraduates)

	**MOCI-J total**	**MOCI-J cleaning**	**MOCI-J checking**	**MOCI-J doubting**	**MOCI-J slowness**	**STAI**	**CES-D**
OCI total	.74***	.55***	.76***	.64***	.60***	.41***	.48***
OCI washing	.59***	.59***	.50***	.44***	.44***	.30**	.32**
OCI checking	.64***	.32***	.71***	.56***	.45***	.31**	.35***
OCI doubting	.63***	.47***	.62***	.68***	.39**	.41***	39***
OCI ordering	.65***	.41***	.63***	.51***	.50***	.34***	.38***
OCI obsessing	.62***	.37**	.65***	.55***	.61***	.45***	.60***
OCI hoarding	.53***	.32**	.51***	.54***	.48***	.29**	.48***
OCI neutralizing	.64***	.44^****^	.64***	.55***	.51***	.37***	.42***

**p < .01. ***p < .001.

CES-D = Center for Epidemiological Studies Depression scale; MOCI-J = Maudsley Obsessive-Compulsive Inventory, Japanese version; OCI-J = Obsessive-Compulsive Inventory, Japanese version; STAI = State-Trait Anxiety Inventory.

### Test-retest reliability

The test-retest interval was 14 days. Reliability estimates were calculated using Pearson’s *r* between scores on the first and second tests. Overall, the pre-post score correlation of the total score of the OCI-J was 0.76 (*p* < .001). The results for the subscales were as follows: Washing, 0.81 (*p* < .001); Checking, 0.80 (*p* < .001); Doubting, 0.77 (*p* < .001); Ordering, 0.72 (*p* < .001); Obsessing, 0.75 (*p* < .001); Hoarding, 0.70 (*p* < .001); and Neutralizing, 0.75 (*p* < .001). These values indicated adequate test-retest reliability.

### Study 2

The purpose of our second study was to investigate whether the OCI-J could successfully discriminate between OCD patients, healthy controls, and anxiety controls (patients with panic disorder).

### Participants and procedure

The clinical participants were recruited from eight outpatient mental health clinics in Japan. Participants were excluded if they were under age 18 or over age 60.

Potential OCD participants (*N* = 48) were diagnosed by a psychiatrist using the OCD criteria of the Diagnostic and Statistical Manual of Mental Disorders, 4th edition, Text Revision (DSM-IV-TR, American Psychiatric Association [34]). Individuals were excluded if they had a comorbid disorder on Axis I or II of the DSM-IV-TR (e.g., other anxiety disorder, major depression, mental retardation, personality disorder, psychotic disorder, dementia, mental retardation, or substance use disorder). In addition, the Japanese version of the self-report Y-BOCS [35] was administered for the participants with OCD; individuals were included if they scored over 16 points (the suggested cut-off for OCD). To confirm that potential OCD participants had no panic disorder or depression symptoms, the Japanese version of the Self-Report PDSS (PDSS-SR-J) [36] and Japanese version of the Beck Depression Inventory, Version II (BDI-II) [37] were administered; individuals were excluded if they scored over the cut-off point (minimal symptoms vs. mild symptoms) of 8 for the PDSS-SR-J [36] and 14 for the BDI-II [38]. The final OCD group consisted of 35 individuals (OCD group; male = 12, female = 23; age range: 20–48 years, *M* = 30.11, *SD* = 3.44).

Potential anxiety control participants (*N* = 36) were diagnosed by a psychiatrist using the DSM-IV-TR criteria for panic disorder. The individuals were excluded if they had a comorbid Axis I or II disorder, but not if they had a comorbid anxiety disorder other than OCD (e.g., a patient with panic disorder and social anxiety disorder). In addition, the PDSS-SR-J [36] was administered; individuals were included if they scored over 8 points. To confirm that they had no OCD or depression symptoms, the self-report Y-BOCS [35] and BDI-II [37] were administered; potential participants were excluded if they scored over the cut-off point of 16 on the self-report Y-BOCS and 14 on the BDI-II. Twenty-two individuals met the above criteria (anxiety control group; male = 9, female = 13; age range: 22–53 years, *M* = 35.60, *SD* = 4.50). The frequency of each principal anxiety disorder was as follows: panic disorder without agoraphobia, 50%; panic disorder with agoraphobia, 32%; panic disorder without agoraphobia and comorbid general anxiety disorder, 9%; panic disorder with agoraphobia and comorbid social anxiety disorder, 5%; and panic disorder with agoraphobia and comorbid post-traumatic stress disorders, 5%.

Non-clinical participants were Japanese university students. Potential participants (*N* = 40) were excluded if they reported a clinical history of mental disorder, head injury, central nervous system diseases, or substance abuse. To confirm that these individuals had no OCD, panic, or depression symptoms, the self-report Y-BOCS, PDSS-SR-J and BDI-II [37] were administered; participants were excluded if they scored over the cut-off point of 16 on the Y-BOCS, 8 on the PDSS-SR-J, and 14 on the BDI-II. Thirty-seven non-clinical Japanese individuals met the inclusion criteria for the healthy control group (male = 17, female = 20; age range: 20–52 years; *M* = 25.61, *SD* = 5.44).

This study was approved by the ethics committee of, the Graduate School of Medicine, Chiba University (receipt number, 1122). All individuals participated on a voluntary basis and gave their written consent before entering the study. Eligible participants completed the OCI-J individually.

### Measurements

In this study, the Self-Report Y-BOCS, PDSS, and BDI-II were administered to confirm whether potential participants met the inclusion or exclusion criteria (see the Participants and procedure section).

#### Japanese version of the OCI (OCI-J)

The OCI–J (see Study 1) was administered to all participants.

#### Japanese version of the Self-Report Y-BOCS

The Japanese version of Self-Report Y-BOCS was developed by Hamaguchi et al. [35]. Participants are first asked to note the presence or absence of 58 obsessions and compulsions from the same general categories as in the interview. They are then asked to circle three main obsessions and three main compulsions. The second part of the report asked participants to focus on these main obsessions and compulsions to answer five questions for each one: time spent, interference, distress, resistance, and control. Consistent with the interview format, participants rated each item on a 0 (*none*) to 4 (*extreme*) scale. The validity and reliability of the Japanese version of the self-report Y-BOCS were confirmed by Hamaguchi et al. [35].

#### Japanese version of the Self-Report PDSS (PDSS-SR-J)

The PDSS, which was originally designed as a face-to-face interview for use in both research and clinical practice [39], was adapted for use as a patient self-report questionnaire [40]. The instrument contains seven items that assess the severity of seven dimensions of panic disorder and its associated symptoms. Responses are given on a five-point Likert-type scale from 0 to 4. The total score ranges from 0 to 28. A higher score is associated with more severe panic symptoms. Japanese version of PDSS-SR (PDSS-SR-J) was developed by Motohisa et al. [36]. Psychometric evaluations indicate that the PDSS-SR-J has adequate internal consistency and construct validity [36].

#### Beck Depression Inventory, Version 2

To measure depressive symptoms, we used the Japanese version of the Beck Depression Inventory, Version 2 (BDI-II [32]). This is a 21-item self-report measure designed to assess symptoms of major depression. Kojima et al. [37] developed the Japanese version. Responses are given on a four-point Likert-type scale from 0 (*not at all*) to 3 (*severely*).

## Results

To compare the total scores of the OCI-J across the groups (OCD, anxious control, and healthy control), we conducted an analysis of variance (ANOVA) with the OCI-J total scores as the dependent variable. There was a significant main effect of Group, *F* (2, 91) = 69.41, *p* < .001, ηp^2^ = .40. We conducted Bonferroni multiple comparisons to compare the OCI-J total scores across groups. The result of the comparison indicated that OCD participants had significantly higher mean total scores on the OCI-J than did the anxious and healthy controls (Table 4).

**Table 4:** Means and SDs of the OCI-J in each group

	**OCD**	**Anxious controls**	**Healthy controls**	
	**N = 35**	**n = 22**	**n = 37**	
	**Mean (SD)**	**Mean (SD)**	**Mean (SD)**	**Group (F)**
OCI-J total score	71.23 (44.12)^a^	40.32 (30.23)^b^	29.98 (22.92)^c^	69.41***
Washing	14.48 (11.32)^a^	9.14 (6.54)^b^	6.04 (6.65)^b^	64.99***
Checking	14.58 (9.54)^a^	9.12 (7.43)^b^	7.09 (6.43)^b^	42.41***
Doubting	5.22 (4.32)^a^	3.01 (2.01)^b^	2.63 (2.71)^b^	24.20***
Ordering	8.60 (7.12)^a^	3.65 (3.46)^b^	3.11 (1.78)^b^	55.26***
Obsessing	15.28 (10.12)^a^	8.89 (6.32)^b^	5.58 (3.36)^c^	79.45***
Hoarding	1.81 (3.12)^a^	1.25 (2.10)^a^	1.52 (1.01)^a^	2.89
Neutralizing	9.72 (7.54)^a^	6.53 (4.34)^b^	2.93 (2.88)^c^	52.65***
Y-BOCS	21.29 (11.56)^a^	7.45 (4.21)^b^	6.22 (5.08)^b^	21.10***
PDSS-SR-J	2.21 (2.02)^b^	9.21 (4.88)^a^	1.21 (2.53)^b^	18.65***
BDI-II	11.45 (3.26)^a^	12.01 (4.07)^a^	10.07 (4.02)^a^	2.11

***Significant main effect of group differences (*p* < .001).

Note: Values on the same line with different superscripts are significantly different from each other, and values with the same superscripts are not significantly different in the multiple comparisons.

OCD = Obsessive Compulsive Disorder patients; OCI-J = Obsessive Compulsive Inventory.

To compare the subscale scores across the groups, we conducted a multivariate analysis of variance (MANOVA) with Washing, Checking, Doubting, Ordering, Obsessing, Hoarding, and Neutralizing subscale scores as the dependent variables. The results showed a significant main effect of Group, *F* (14, 172) = 10.99, *p* < .001, ηp^2^ = .47.

We also conducted one-way ANOVAs and Bonferroni multiple comparisons to compare the questionnaire scores across groups. The results of these comparisons are given in Table 4.

Our results indicated that the OCD group differed from both comparison groups on the Washing (*F* (2, 91) = 64.99, *p* < .001), Checking (*F* (2, 91) = 42.41, *p* < .001), Doubting (*F* (2, 91) = 24.20, *p* < .001), Ordering (*F* (2, 91) = 55.26, *p* < .001), Obsessing (*F* (2, 91) = 79.45, *p* < .001), and Neutralizing (*F* (2, 91) = 52.65, *p* < .001) subscales. However, the Hoarding subscale scores did not differ significantly between OCD group and the comparison groups (*F* (2, 91) = 2.89, *p* < .18).

Multiple comparisons indicated that OCD participants had significantly higher mean total scores on the OCI-J and on most of the subscales (Washing, Checking, Doubting, Ordering, Obsessing, and Neutralizing) than did the anxious and healthy controls. Furthermore, the anxious control group had significantly higher mean total score on the OCI-J, as well as on the Obsessing and Neutralizing subscales, than did the non-clinical control participants.

## Discussion

The aim of this study was to validate a Japanese version of the OCI in both non-clinical and clinical samples. Both the internal consistency and the test-retest reliability of the OCI-J were confirmed using non-clinical participants. Cronbach’s alpha coefficients for the full scale and the subscales were consistent with the previous studies of Foa et al. [12] and Simonds et al. [21]. We can safely say that the OCI-J has sufficient coherence because these coefficients were high (ranging from 0.80 to 0.96 in Cronbach’s alpha). In addition, the total score of the OCI-J and the subscales demonstrated satisfactory test-retest reliability (ranging from = 0.70 to 0.81 in correlation), which is consistent with previous research (Foa et al. [12], range = 0.68 to 0.89; Simonds et al. [21], range = 0.71 to 0.88). These results indicate that the OCI-J has acceptable reliability in measuring OCD symptoms in a Japanese setting.

Moreover, we observed a strong correlation between the OCI-J and MOCI-J total scores (*r* = 0.74). The OCI-J subscales were also significantly correlated with the MOCI-J subscales: the correlation coefficient between the OCI-J Washing subscale and the MOCI-J Cleaning subscale was 0.59, between Checking subscales was 0.71, and between the Doubting subscales was 0.68. These correlations with the MOCI-J confirm acceptable convergent validity of the OCI-J in measuring OCD symptoms in a Japanese setting. Regarding the relationship with other psychopathologies, the OCI-J total score was found to correlate with depression (CES-D; *r* = 0.48) and anxiety (STAI; *r* = 0.41), as were the seven subscales of the OCI-J. These results indicate that the OCI-J has acceptable discriminative validity in measuring OCD symptoms because the correlation coefficients between the OCI-J and MOCI-J were higher than between the OCI-J, CES-D, and STAI.

The correlation between the Obsessing subscale on the OCI-J and depression scores of the CES-D in the student sample were higher than the relationship of the other subscales to depression. A previous study using an Iranian student sample also found that the Obsessing subscale of OCI correlated with depression measured by BDI-II more strongly than with the other subscales [41]. These results mean that the non-clinical students who reported obsessing symptoms may have depressive symptoms more often than students who had other OCD symptoms.

Our study also showed that the OCI-J accurately discriminated between patients with OCD and anxious or healthy controls. As predicted, participants with OCD had significantly higher mean scores on the OCI-J total score and the Washing, Checking, Doubting, Ordering, Obsessing, and Neutralizing subscales than the anxious and healthy controls. The non-significant finding for the Hoarding subscale is consistent with the original OCI validation study [12], which found that the scores of patients with OCD were no more elevated on this subscale than were the scores of non-clinical participants. In the study of the OCI-R in China, the Hoarding subscale demonstrated poor discrimination between OCD patients and anxiety patients, as well as between OCD patients and student participants [24]. Thus, Hoarding may be a minor symptom in OCD patients. Recent studies [42,43] have found ample evidence that hoarding should not be conceptualized only as an OCD symptom. Indeed, hoarding is considered a stand-alone disorder in the DSM-5 [44,45], which is supported by our findings. The Hoarding subscale of the OCI-J should be interpreted cautiously due to the evidence of its inability to discriminate groups in a Japanese sample.

Our study suggests that the OCI-J has adequate construct validity because the convergent and discriminant validity were verified. Construct validation can be established by examining whether a construct of interest is related to other variables with which it should be associated theoretically [46]. However, full confirmation of the construct validity requires an assessment of the factor structure of the OCI-J, which was not done in the present study.

The present study used clinical and non-clinical groups to verify the psychometric properties of the OCI-J. Non-clinical samples were used to confirm the psychometric properties of the OCI-J because such samples may be less selective than patient samples and, therefore, provide an opportunity to study etiological factors [21,47]. In addition, non-clinical samples are often used in OCD research, and several studies have demonstrated continuity between non-clinical and OCD samples in the characteristics of their obsessions and compulsions [48].

There was a considerable difference in the scores of anxious controls in this study and those in the original study by Foa et al. [12]. The OCI-J total mean score of anxious controls in this study was 40.32 (SD = 30.23); however, in Foa et al., the OCI total mean was 28.81 (SD = 22.1) for generalized social phobia and 35.70 (SD = 26.0) for PTSD. These differences might be due to the source (outpatient mental health clinics) and relatively small sample size of the anxiety control participants and the potential cultural impact of reporting OCD symptoms in our current sample. Furthermore, the characteristics of the anxious control participants were different in the present study (panic disorder) from those in Foa et al.’s study (generalized social phobia or PTSD).

Compared to the Y-BOCS, the OCI-J has some advantages: trained interviewers are not required, it can be used to assess OC thoughts and behaviors in the general population, and it is a more comprehensive instrument. However, in terms of subjective distress of OCD patients, Y-BOCS scores are less affected by OCD subtypes than the OCI self-report scores.

## Conclusion

Our findings demonstrated that the OCI-J exhibits satisfactory psychometric properties. Thus, the OCI-J appears to be an ecologically and culturally valid measure of OCD symptoms. The availability of the OCI-J provides researchers with an additional measure for assessing the severity of OCD symptoms and, given the popularity of the OCI in other countries, could facilitate cross-cultural comparison in the near future.

### Limitations and future research

This study has a number of limitations that must be addressed. First, we did not investigate the relationship between the OCI-J and other self-report measures of OCD, such as the Japanese version of Padua Inventory [48,49], or clinician-administered scales, such as the Y-BOCS [6]. These comparisons would be helpful to fully establish concurrent validity. Nevertheless, we believe that our use of the MOCI-J was suitable for this study because the MOCI-J was used to assess the concurrent validity of the OCI [21] and the Chinese version of the OCI-R [23] in previous studies.

The small clinical sample size and lack of a standardized semi-structured interview, such as the SCID-I/P [50], to confirm clinical diagnoses in the clinical sample, must be noted as a limitation. Furthermore, we did not investigate the factorial validity of the OCI due to the small sample size. The original study reporting the development of the OCI [12] suggested that the OCI has a seven-factor structure, while Sica et al. [27] proposed a five-factor structure based on the factor analysis of a non-clinical sample. Thus, the factor structure of the OCI appears to vary by study. There has been no research on the factorial validity of the OCI using a clinical sample in an Eastern culture. Further studies using clinical samples are required to confirm if the factor structure of the OCI is consistent across Western and Eastern cultures or if the OCI-J has a different factor structure that is more reflective of the Japanese culture.

Nonetheless, the present study is a first step in confirming the validity of the OCI-J. We plan to study the psychometric properties of the OCI-J further, including performing factor analysis using Japanese clinical samples. In addition, we hope that our results will contribute to the development of a short version of the OCI (i.e., the OCI-R) for use in Japanese populations.

## Abbreviations

OCD: Obsessive-compulsive disorder; OCI: Obsessive-compulsive inventory; MOCI: Maudsley obsessive-compulsive inventory; Y-BOCS: Yale-brown obsessive compulsive scale; CES-D: Center for epidemiological studies depression scale; STAI: State-trait anxiety scale.

## Competing interests

The authors declare that they have no competing interests.

## Authors’ contributions

RI designed and managed the study, performed the statistical analyses, and drafted the manuscript. OK and ES supervised the overall conduct of the study. All authors read, critically revised, and approved the final manuscript.
